# What We Want to Know

**Published:** 1897-01

**Authors:** 


### WHAT WE WANT TO KNOW.

ONE of our esteemed contemporaries in a recent issue exhibited in its reading columns the deepest concern and regret at the knowledge of the fact that one of our strong veterinary centres of learning had failed to establish in one of her offspring sufficiently high ethical culture to save him from endeavoring to flood the market with a proprietary remedy of such untold value that a standing wager was made as an evidence of its boasted merits as a curative agent. The same journal has recently opened wide its advertising pages for the sale of such preparations, and one is led to wonder which may prove the greater evil: to prepare such articles of commerce or to allow them to be advertised in scientific journals, which gives them an apparent if not real stamp of merit—an indirect approval that sporting or live-stock journals do not give to these remedies in accepting their advertisements.

---

THE JOURNAL craves the indulgence of its contributors for a little while. We find ourselves so overwhelmed with material that much of it, though important and of great value by its early publication, must be postponed for future issues. We are very thankful to our many friends for this best of all testimony of the interest thus exhibited in making the JOURNAL full and complete in every direction, and we shall endeavor to make the most of your generosity for the good of our profession.

---

THE appointment of Dr. Austin Peters on the Massachusetts Cattle Commission, in place of Dr. F. H. Osgood, resigned, is the recognition of one who has spent long and earnest years in the study of infectious and contagious diseases of the lower animals. He has served in official place under the Massachusetts Society for the Promotion of Agriculture, and was selected by the State Board of Health of New York in its endeavor to arrive at a knowledge of the extent of tuberculosis in the Empire State. The subject of tuberculosis is a familiar one in every aspect. From early study, observation, and investigation, Dr. Peters possesses a trained mind, is a thorough professional man, and his work will be a conscientious and painstaking duty for the people in whose interest he has now assumed a position of great responsibility. We wish for him in his new place the utmost success in all his efforts, and the JOURNAL will follow the pathway of his work with zeal and patience. He will now take up the most important position in our whole land on the subject of tuberculosis, and there will be the utmost interest in all that he does for his own people, who have become leaders in all progessive movements to stamp out dangerous and costly infectious and contagious diseases of live-stock. The people throughout the civilized world will be benefited by valuable lessons to be learned from the fruits of the labor which he will direct. With large means and power to be commanded; with no geographical limits too large for a thorough plan of action; no physical barriers that cannot be overcome or controlled; with ground better tilled by the conscientious work of his predecessors than anywhere in our land, we bid him welcome and wait with patience the fruition of his labors.

